# *Triatoma infestans* Bugs in Southern Patagonia, Argentina

**DOI:** 10.3201/eid1605.091260

**Published:** 2010-05

**Authors:** Romina V. Piccinali, Delmi M. Canale, Alejandra E. Sandoval, Marta V. Cardinal, Oscar Jensen, Uriel Kitron, Ricardo E. Gürtler

**Affiliations:** Universidad de Buenos Aires, Buenos Aires, Argentina (R.V. Piccinali, M.V. Cardinal, R.E. Gürtler); Coordinación Nacional de Control de Vectores, Córdoba, Argentina (D.M. Canale); Secretaría de Salud de Chubut, Chubut, Argentina (A.E. Sandoval, O. Jensen); Emory University, Atlanta, Georgia, USA (U. Kitron)

**Keywords:** Triatoma infestans, Trypanosoma cruzi, passive dispersal, mitochondrial cytochrome oxidase I, mtCOI, phylogenetic analysis, surveillance, Argentina, letter

**To the Editor:** *Triatoma infestans* bugs, the main vector of Chagas disease, historically occupied a large area from northeastern Brazil to Chubut Province in Patagonia, Argentina (*1*). Large-scale insecticide spraying during the 1980s and 1990s reduced its geographic range and abundance and interrupted transmission of *Trypanosoma cruzi*, mainly in Uruguay, Chile, and Brazil (*2*). However, *T*. *infestans* and transmission of *T*. cruzi persist in the Gran Chaco, a large ecoregion in Argentina, Bolivia, and Paraguay (*3*).

Chubut Province has historically been an area with no risk for vector-mediated transmission of *T*. *cruzi*; only its extreme northern region was categorized as having a low transmission risk (*4**,**5*). However, increased immigration from disease-endemic rural areas in Argentina and Bolivia into Chubut has raised concerns about accidental introduction of *T*. *infestans* in travelers’ luggage (*1*) and establishment of a transmission cycle.

In January 2007, we conducted a province-wide survey of 21 villages in Chubut Province previously infested with *T*. *infestans* bugs by using 0.2% tetramethrin as a dislodgant agent (1 person-hour/house); no *T*. *infestans* bugs were detected (Appendix Figure). Only *T*. *patagonica* bugs were found in 11% of peridomestic structures, and none were infected with *T*. *cruzi*. In June 2007, a *T*. *infestans*–like bug was found in a primary healthcare center in Comodoro Rivadavia (45°51′S, 67°28′W), a city in southern Chubut Province (Appendix Figure). Healthcare center staff reported visits by immigrants from Bolivia a few days before this finding and suspected them to be the source. The bug was identified morphologically as a *T*. *infestans* female and it laid 6 eggs. PCR amplification of kinetoplast DNA showed that it was not infected by *T*. *cruzi*.

DNA sequence analysis is useful for investigating evolutionary history and population structure within Triatominae (*6*). *T*. *infestans* bugs from Bolivia and Argentina showed genetic differences for nuclear (*7*) and mitochondrial markers (*6*), including mitochondrial cytochrome oxidase I (mt*COI*) (*8*). We used our mt*COI* haplotype database, which includes published (*8*) and new domestic, peridomestic, and sylvatic *T*. *infestans* from 65 locations in 13 provinces in Argentina (n = 346) and 3 departments in Bolivia (n = 144), to analyze the mt*COI* sequence of the bug found in southern Patagonia and determine if it could be assigned to a known haplotype from Bolivia or Argentina. We investigated phylogenetic relationships with other haplotypes by using neighbor-joining and Bayesian approaches.

Our mt*COI* database included 53 haplotypes: 42 were found in Argentina, 9 in Bolivia, and 2 in both countries (Figure). The bug from southern Patagonia had haplotype *x*, which has been found in only 3 western or southern provinces in Argentina (San Juan, San Luis, and Rio Negro) (*8*; Appendix Figure).

**Figure:**
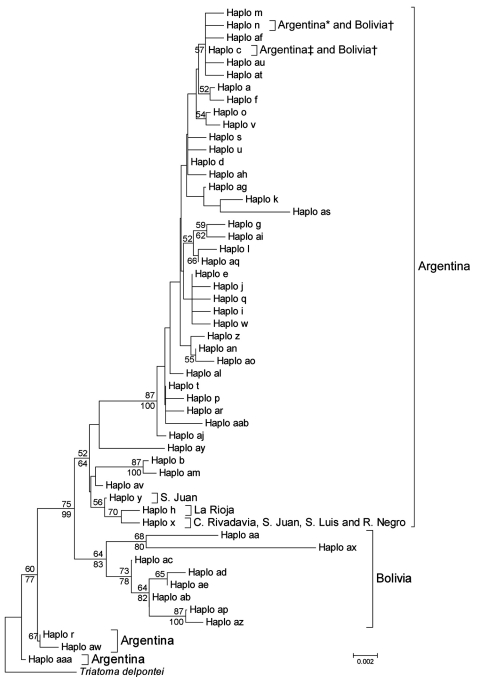
Phylogenetic relationships between mitochondrial cytochrome oxidase I gene haplotypes of *Triatoma infestans* from Argentina and Bolivia. The neighbor-joining tree was constructed by using MEGA 4.1 (www.megasoftware.net) and bootstrap values (based on 1,000 replications) >50% are shown above the branches. A Bayesian maximum clade credibility tree was similar, and clade posterior probabilities >50% are shown below the branches of the neighbor-joining tree. MRBAYES 3.1 (http://mrbayes.csit.fsu.edu) default priors were assumed and run for 4 million generations. Convergence of the Markov chain Monte Carlo analysis was investigated with the SD of split frequencies and diagnostics implemented in AWTY (http://ceb.csit.fsu.edu/awty). The model of evolution (Hasegawa-Kishino-Yano + invariable sites+ Γ [HKY + I + Γ]) was chosen with Mrmodeltest 2.3 (www.abc.se/~nylander). Because MEGA 4.1 does not support HKY; the more inclusive Tamura-Nei method (www.megasoftware.net/WebHelp/part_iv___evolutionary_analysis/computing_evolutionary_distances/distance_models/nucleotide_substitution_models/hc_tamura_nei_distance.htm) was used for the neighbor-joining analysis. Haplotypes *al*, *an*, *ao*, *ap*, *aq*, *at*, *au*, *ax*, *az*, *aaa*, and *aab* are reported. DNA sequences are available in GenBank (accession nos. EF451005-EF451041, FJ439768, FJ811845–8, and GQ 478993–GQ 479005). *Two provinces in Argentina; †Tarija, Bolivia; ‡10 provinces in Argentina. Scale bar indicates nucleotide substitutions per site.

Results of phylogenetic analyses were congruent (Figure). The neighbor-joining tree showed that haplotype *x* formed a cluster with haplotype *h* (Argentina) and haplotypes from Bolivia clustered in 3 other groups: 1) two groups with bootstrap values >70% (one with haplotypes *at*, *n*, *c*, and 33 haplotypes from Argentina, and the other with haplotypes *ab*, *ac*, *ad*, *ae*, *ap*, and *az*); and 2) one group with a bootstrap value of 68% (haplotypes *ax* and *aa*). The Bayesian tree showed that haplotypes from Bolivia were arranged in 2 well-supported clades (posterior probabilities >83%) and that haplotype *x* was not included within any of them. Thus, haplotype *x* of the bug from southern Patagonia was found only in Argentina and was not closely related to haplotypes from Bolivia.

We investigated the geographic origin of non-native putative attendees of the healthcare center in San Cayetano. These persons were immigrants from Bolivia and from northern (Salta and Jujuy), western (Mendoza, San Juan, and San Luis), and southern Argentina (Rio Negro), i.e., from the 3 putative sources of the bug. These immigrants typically pay extended visits to their home towns at least once a year and transport luggage in which the bug could have traveled. In 2006, San Juan had the highest levels of domestic and peridomestic infestation with *T*. *infestans* (35% and 21%, respectively), including urban infestation (*9*). Mendoza (not in our database) had considerable domestic and peridomestic infestations (both 7%), and San Luis (0.5% and 5.3%, respectively) and Rio Negro (both <0.1%) had low infestations in 2001 (*4*) and thereafter (C. Spillmann, unpub. data). Bolivia, Salta, and Jujuy are excluded as potential sources of the bug because haplotypes closely related to haplotype *x* were not found in these places. Active dispersal from a local source can be ruled out because there is no precedent for *T*. *infestans* in Comodoro Rivadavia, and the mean temperature in June (8°C) is below the known threshold for flight initiation (23°C) (*10*).

Our results show that molecular phylogenetics can identify passive transport of insects into areas where a disease is not endemic and rule out putative sources supported only by circumstantial evidence. Our findings reinforce the need for sustained and coordinated vector surveillance and control at a regional level (*3*).

## Supplementary Material

Appendix Figure Location of Comodoro Rivadavia (Chubut) and mitochondrial cytochrome oxidase I haplotype frequencies among *Triatoma infestans* bugs in provinces in Argentina and departments in Bolivia. Colors and patterns in circles indicate frequencies of each haplotype in an area. The haplotype of the bug from southern Patagonia (*x*) is indicated in red. Shared haplotypes between Argentina and Bolivia are indicated in blue (haplotype *c*) and green (haplotype *n*). Yellow areas indicate provinces surveyed. Gray shading indicates areas of Chubut Province where *T. infestans* bugs were not found in 2007. S. C., Santa Cruz; Sgo., Santiago.
