# Supplementary figures and images for: Triatoma infestans Bugs in Southern Patagonia, Argentina
Source: Emerg Infect Dis. 2010 May;16(5):887–9. doi: 10.3201/eid1605.091260 (PMC2954526; doi:10.3201/eid1605.091260)

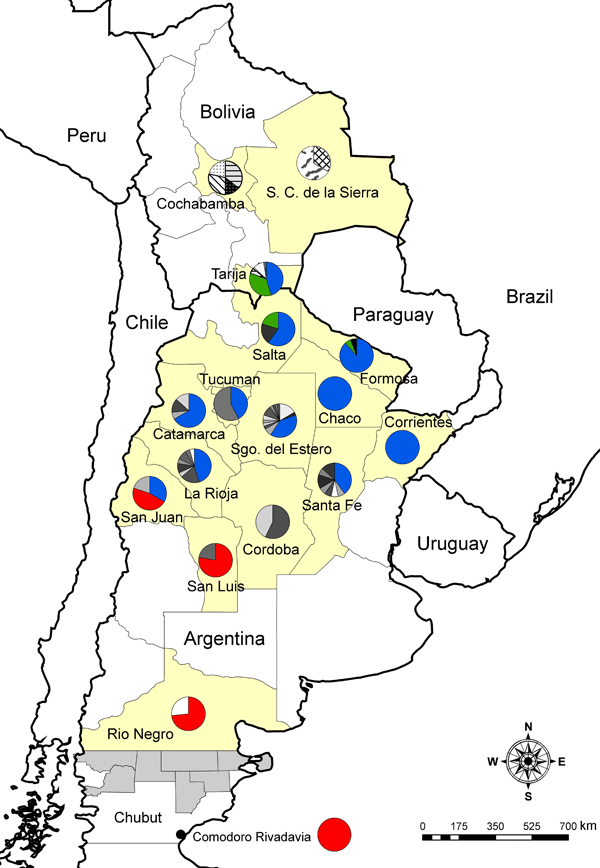

Supplement: Appendix Figure — Location of Comodoro Rivadavia (Chubut) and mitochondrial cytochrome oxidase I haplotype frequencies among Triatoma infestans bugs in provinces in Argentina and departments in Bolivia. Colors and patterns in circles indicate frequencies of each haplotype in an area. The haplotype of the bug from southern Patagonia (x) is indicated in red. Shared haplotypes between Argentina and Bolivia are indicated in blue (haplotype c) and green (haplotype n). Yellow areas indicate provinces surveyed. Gray shading indicates areas of Chubut Province where T. infestans bugs were not found in 2007. S. C., Santa Cruz; Sgo., Santiago. [file 09-1260_appF-s1.gif]
